# Artificial Intelligence Versus Human Expertise in Implant Treatment Planning: A Retrospective Cone-Beam Computed Tomography-Based Comparative Study

**DOI:** 10.7759/cureus.109410

**Published:** 2026-05-21

**Authors:** Priyanka Jadhav, Shruthi P, Shveta Jain, Hafsa Parveen, Hariyabbe Rangaswamy Likhithaswamy, Syed Ashfaq Ur Rahman

**Affiliations:** 1 Department of Prosthodontics, Bharati Vidyapeeth (Deemed to be University) Dental College and Hospital, Sangli, IND; 2 Department of Conservative Dentistry and Endodontics, Government Medical College, Nalgonda, IND; 3 Department of Prosthodontics, Jain Dental and Oral Care Centre, Dehradun, IND; 4 Department of Oral and Maxillofacial Surgery, Dr. Godvine's Clinique, Hyderabad, IND; 5 Department of Oral Pathology, Sri Siddhartha Dental College, Heggere, IND; 6 Department of Oral and Maxillofacial Surgery, Kamineni Institute of Dental Sciences, Akkinepallivari Lingotam, IND

**Keywords:** artificial intelligence, artificial intelligence versus human expertise, comparative study, cone-beam computed tomography, dental implants, implant treatment planning, prosthodontics, treatment planning

## Abstract

Introduction

Accurate implant treatment planning is essential to achieve optimal functional and esthetic outcomes in modern dentistry. With the increasing integration of digital technologies, artificial intelligence (AI) has emerged as a promising tool for enhancing precision, consistency, and efficiency in clinical decision-making. However, evidence comparing AI-assisted planning to conventional manual approaches remains limited. This study aimed to compare the accuracy, efficiency, and reliability of AI-assisted implant treatment planning with manual clinician-based assessment, using cone-beam computed tomography (CBCT) data.

Materials and methods

This retrospective cross-sectional study included 30 CBCT scans of patients who underwent dental implant placement. Each dataset was analyzed using AI-assisted planning with Simplant Pro (Materialise NV, Leuven, Belgium; distributed by Dentsply Sirona, Charlotte, North Carolina, USA) and manual assessment by a blinded clinician. The evaluated parameters included bone height, buccolingual width, angular deviation, linear deviation, planning time, and clinician confidence. Postsurgical radiographs were used as reference standards. All the statistical analyses were performed. Paired t-tests and Wilcoxon signed-rank tests were applied, with p<0.05 considered statistically significant.

Results

AI demonstrated slightly lower bone height (12.06 ± 1.62 mm vs 12.36 ± 1.78 mm) and width (7.18 ± 0.82 mm vs 7.55 ± 0.98 mm) compared to manual assessment (p<0.05). Significantly lower angular deviation (1.90 ± 0.83° vs 3.99 ± 1.09°​​​​​​​) and linear deviation (0.43 ± 0.15 mm vs 1.01 ± 0.38 mm) were observed with AI (p<0.0001). Planning time was significantly reduced (8.5 ± 2.1 vs 21.4 ± 5.7 minutes), and clinician confidence was higher with AI (82.1 ± 9.2 vs 68.9 ± 11.0; p<0.001). The intraclass correlation showed excellent agreement with the bone measurements (Intraclass Correlation Coefficient or ICC>0.80).

Conclusion

AI-assisted implant planning demonstrated superior accuracy, efficiency, and consistency compared to manual methods. It can serve as a reliable adjunct to enhance clinical decision making and optimize treatment outcomes. Clinician acceptance of nanorobotic interventions is scenario-dependent, with higher preference in precision-based and minimally invasive cases. However, concerns regarding safety and procedural control continue to limit broader adoption. Prior experience with advanced technologies significantly influences acceptance. Wider clinical use will depend on stronger evidence and improved training.

## Introduction

Dental implant therapy has become a predictable and widely accepted treatment modality for the replacement of missing teeth, with success increasingly dependent on meticulous pre-operative planning. Implant treatment planning requires the integration of multiple clinical variables, including anatomical considerations, prosthetic requirements, patient-specific factors, and risk assessment [1,2]. Traditionally, such planning has relied on a clinician’s knowledge, experience, and interpretative skills, which can vary significantly across different levels of training and expertise.

In recent years, artificial intelligence (AI) has emerged as a transformative tool in healthcare and dentistry. AI-driven systems have demonstrated the ability to process complex clinical information, generate diagnostic suggestions, and assist in decision making [3,4]. These systems offer potential advantages, such as rapid information processing, consistency in responses, and accessibility [5]. However, concerns remain regarding their clinical reliability, contextual understanding, and ability to replicate nuanced human judgments in complex treatment planning scenarios [6].

Despite the growing interest in AI applications, there is limited evidence evaluating their performance in comparison with clinicians in implant treatment planning, particularly in non-radiographic, scenario-based contexts [7]. Understanding whether AI can match or approximate clinician decision making is essential before integrating such tools into routine clinical workflows. Additionally, comparing AI performance across different levels of dental expertise may provide insight into its role as an adjunctive educational and clinical support tool [8].

This study aimed to evaluate and compare the knowledge and decision-making accuracy of AI and dental professionals in implant treatment planning using standardized clinical scenarios. The objectives were to assess the accuracy of responses generated by AI and clinicians, compare performance across different levels of clinical experience, and determine the agreement and variability in treatment planning decisions between AI and human participants.

## Materials and methods

Study design and setting

This retrospective, cross-sectional, observational study was conducted at the Department of Prosthodontics, Bharati Vidyapeeth (Deemed to be University) Dental College and Hospital, Sangli, India, from April 2025 to May 2025. This study utilized archived cone-beam computed tomography (CBCT) datasets, along with corresponding post-surgical radiographic records obtained from January 2021 to December 2024. As no direct patient interaction or intervention was involved, the study design minimized the clinical risk and followed established observational research principles. Given the retrospective nature of the study and the use of previously recorded clinical data, a waiver for ethical approval and informed consent was given. All CBCT datasets were anonymized before analysis to ensure confidentiality and compliance with ethical standards.

Sample size determination

Sample size estimation was performed using G*Power (version 3.1, Heinrich-Heine-Universität Düsseldorf, Düsseldorf, Germany). Based on a paired t-test model with a predetermined alpha level of 0.05, statistical power of 80%, and a moderate effect size of 0.50, derived from previous literature, the minimum required sample size was calculated to be 27 CBCT scans [9]. The final sample size was increased to 30 CBCT scans to compensate for potential exclusions owing to inadequate scan quality.

Study sample and eligibility criteria

Thirty CBCT scans of patients who had undergone dental implant placement were included in the study. Only cases with complete clinical documentation and both preoperative and postoperative imaging were selected to ensure the accuracy of the comparison. Patients aged ≥18 years with available preoperative CBCT scans and corresponding postoperative imaging findings were included in the study. Patients with poor-quality scans, significant imaging artifacts, grafted sites involving non-autogenous materials, systemic conditions affecting bone metabolism, or incomplete records were excluded from the study to reduce confounding variables and maintain data reliability.

Study groups and assessment protocol

A within-subject comparison design was adopted, wherein each CBCT scan was evaluated using both the AI-assisted and manual planning approaches. For the AI-assisted assessment, CBCT datasets were analyzed using Simplant Pro (Materialise NV, Leuven, Belgium; Dentsply Sirona, Charlotte, North Carolina, USA). The software generated automated measurements including bone height, buccolingual width, bone density, and suggested implant dimensions and angulation. For the manual assessment, the same CBCT datasets were independently evaluated by a blinded and calibrated clinician with experience in implant dentistry. The clinician performed measurements and treatment planning using standard implant planning software, without access to AI-generated outputs, thereby minimizing bias. Postsurgical CBCT scans or periapical radiographs were used as reference standards to evaluate the accuracy of both methods.

Variables and outcome measures

Primary outcome measures included bone morphometric parameters, such as available bone height and buccolingual width, as well as accuracy of implant positioning assessed through angular and linear deviation from the actual post-surgical implant position. Secondary outcome measures included planning efficiency as assessed by the time taken for planning (in minutes). Clinician confidence was assessed using a 100-mm visual analog scale (VAS), where zero indicated no confidence and 100 indicated maximum confidence [10]. Implant dimension selection accuracy (length and diameter) was also evaluated as an additional performance indicator.

Data collection procedure

All CBCT scans were retrieved in the Digital Imaging and Communications in Medicine (DICOM) format and analyzed under standardized conditions. AI-based planning was first performed to avoid operator bias, followed by manual evaluation by a blinded clinician. The planning time for each method was recorded using a digital stopwatch. All measurements and outputs were systematically documented on a structured data collection sheet for subsequent analyses.

Statistical analysis

All collected data were compiled and analyzed using IBM SPSS Statistics for Windows, Version 26 (Released 2019; IBM Corp., Armonk, New York, United States). Descriptive statistics are expressed as mean and standard deviation for continuous variables and as frequencies with percentages for categorical variables. Normality of the data distribution was assessed prior to inferential testing with Shapiro Wilk test. For normally distributed variables, paired t-tests were used to compare the AI and manual methods. Non-parametric variables, such as planning time and clinician confidence, were analyzed using the Wilcoxon signed-rank test. The agreement between the two methods was evaluated using the intraclass correlation coefficient (ICC) and Bland-Altman plot. Statistical significance was set at p<0.05.

## Results

Thirty CBCT scans were included in the analysis. The mean age of patients was 48.6 ± 11.3 years, with a slight male predominance. Most implant sites were located in the posterior mandible, followed by the posterior maxilla. The mean voxel size was 0.20 ± 0.05 mm, indicating high-resolution imaging suitable for implant planning (Table 1).

**Table 1:** Baseline characteristics of included cone-beam computed tomography (CBCT) scans (n=30) Age is presented as mean ± standard deviation (SD); categorical variables are presented as frequency (percentage); voxel size expressed in millimeters (mm).

Characteristic		Data value
Patient age (years)	Mean ± SD	48.60 ± 11.30
Sex	Male, n (%)	17 (56.7%)
	Female, n (%)	13 (43.3%)
Implant site region	Posterior mandible	22 (73.3%)
	Posterior maxilla	8 (26.7%)
Scan voxel size (mm)	Mean ± SD	0.20 ± 0.05

Descriptive analysis revealed that AI-assisted planning yielded slightly lower mean values for bone height (12.06 ± 1.62 mm) and buccolingual width (7.18 ± 0.82 mm) compared to manual assessment (12.36 ± 1.78 mm and 7.55 ± 0.98 mm, respectively). However, these differences were minimal and clinically negligible. In contrast, substantial differences were observed in accuracy-related parameters, with AI demonstrating significantly lower angular deviation (1.90 ± 0.83° vs 3.99 ± 1.09°) and linear deviation (0.43 ± 0.15 mm vs 1.01 ± 0.38 mm). Additionally, AI showed markedly reduced planning time (8.5 ± 2.1 minutes vs 21.4 ± 5.7 minutes) and higher clinician confidence scores (82.1 ± 9.2 vs 68.9 ± 11.0). The findings are summarized in Table 2.

**Table 2:** Descriptive statistics for measured parameters in AI and manual assessment (n=30) Values are expressed as mean ± standard deviation (SD); angular deviation in degrees (°); linear deviation in millimeters (mm); planning time in minutes; confidence measured using visual analog scale (VAS: 0–100).

Parameter	AI software (Mean ± SD)	Manual assessment (Mean ± SD)	Minimum	Maximum
Bone height (mm)	12.06 ± 1.62	12.36 ± 1.78	8.9	15.8
Bucco-lingual width (mm)	7.18 ± 0.82	7.55 ± 0.98	5.6	9.1
Angular deviation (°)	1.90 ± 0.83	3.99 ± 1.09	0.3	6.2
Linear deviation (mm)	0.43 ± 0.15	1.01 ± 0.38	0.1	1.9
Planning time (min)	8.50 ± 2.10	21.40 ± 5.70	4.8	32.6
Clinician confidence (VAS)	82.10 ± 9.20	68.90 ± 11.00	55.0	100.0

Inferential statistical analysis using paired t-tests demonstrated statistically significant differences between the AI and manual methods across all the evaluated parameters. AI measurements were significantly lower for bone height (mean difference = −0.30 mm, p=0.011) and buccolingual width (mean difference = −0.37 mm, p=0.001), although these differences were small. More importantly, highly significant reductions were observed in angular deviation (mean difference = −2.09°, p<0.0001) and linear deviation (mean difference = −0.57 mm, p<0.0001), indicating superior positional accuracy of AI-assisted planning. Table 3 presents the results.

**Table 3:** Paired comparison of morphometric and accuracy parameters between AI and manual methods (paired t-test) Mean difference calculated as AI − manual; SD: standard deviation; df: degree of freedom; CI: confidence interval; negative values indicate lower measurements by AI; *p<0.05 considered statistically significant.

Parameter	Mean difference (AI − Manual)	SD of the difference	t-value (df = 29)	p-value	95% CI of the difference
Bone height (mm)	−0.30	0.606	−2.725	0.011*	−0.527 to −0.074
Bucco-lingual width (mm)	−0.37	0.571	−3.579	0.001*	−0.586 to −0.160
Angular deviation (°)	−2.09	1.055	−10.863	<0.001*	−2.487 to −1.699
Linear deviation (mm)	−0.57	0.452	−7.008	<0.001*	−0.747 to −0.410

Non-parametric analysis using the Wilcoxon signed-rank test revealed that AI-assisted planning was significantly faster and associated with higher clinician confidence. The median planning time for AI was 8.2 minutes compared to 21.0 minutes for manual assessment (p<0.0001). Similarly, clinician confidence scores were significantly higher for AI (median 83.5 vs 70.0, p=0.0002). The comparisons are presented in Table 4.

**Table 4:** Comparison of planning time and clinician confidence between AI and manual methods (Wilcoxon signed-rank test) Values expressed as median (interquartile range, IQR); VAS: visual analog scale; *p<0.05 considered statistically significant.

Parameter	AI median (IQR)	Manual median (IQR)	W-statistic	p-value	Interpretation
Planning time (minutes)	8.2 (6.9–9.8)	21.0 (17.2–25.3)	25.0	<0.001*	AI significantly faster
Clinician confidence (VAS)	83.5 (76.0–89.3)	70.0 (62.5–77.0)	53.5	0.002*	AI significantly higher

Agreement analysis using ICC demonstrated excellent agreement between the AI and manual methods for bone height (ICC=0.937) and buccolingual width (ICC=0.805). Angular deviation showed moderate agreement (ICC=0.401), whereas linear deviation demonstrated poor agreement (ICC=−0.218), suggesting systematic differences in positional accuracy between the two methods. The findings are summarized in Table 5.

**Table 5:** Intraclass correlation coefficient (ICC) analysis for agreement between AI and manual assessment CI: confidence interval; interpretation based on standard thresholds (≥0.75 excellent, 0.60–0.74 good, 0.40–0.59 moderate, <0.40 poor). The 95% CI for linear deviation is reported as "<0" because the confidence interval lies entirely below zero, consistent with the negative ICC and F‑ratio <1, indicating poor agreement due to systematic bias.

Parameter	ICC value	95% CI (approximately)	F-ratio	Interpretation
Bone height (mm)	0.937	0.872 – 0.968	30.9	Excellent
Bucco-lingual width (mm)	0.805	0.623 – 0.900	9.3	Excellent
Angular deviation (°)	0.401	0.085 – 0.637	2.3	Moderate
Linear deviation (mm)	−0.218	<0	0.6	Poor (systematic bias)

**Figure 1:**
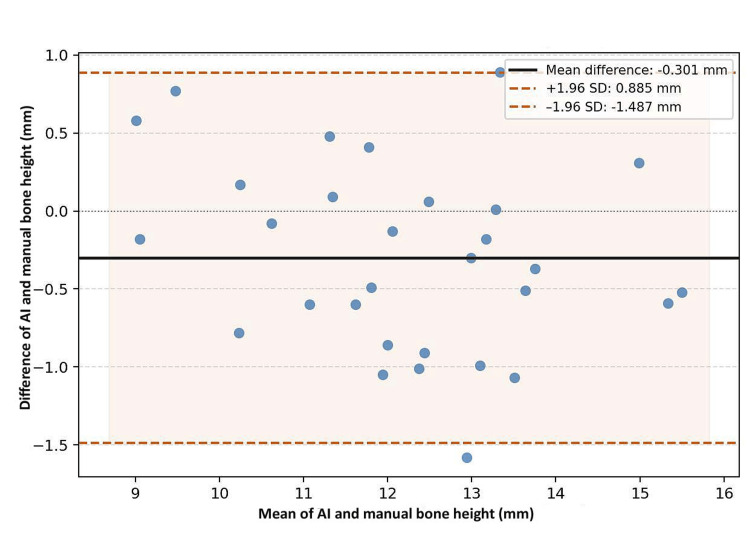
Bland-Altman plot illustrating bone height (mm) by AI software vs manual assessment

The Bland-Altman plot for bone height comparison between AI and manual assessment (Figure 1) demonstrated good agreement, with most data points falling within ±1.96 standard deviation limits.

The mean bias was −0.301 mm, indicating that the AI measurements were slightly lower than the manual measurements without evidence of proportional bias.

The comparison of implant dimension selection accuracy showed that AI-assisted planning achieved a higher number of correct selections, particularly for the implant length. Statistical analysis using McNemar’s test revealed a significant difference in the accuracy of implant length selection (p=0.027), favoring AI. Overall, AI demonstrated improved accuracy, efficiency, and consistency compared with manual implants.

## Discussion

The present study evaluated the accuracy, efficiency, and agreement of AI-assisted implant planning with manual clinician-based assessments using retrospective CBCT data. The findings demonstrated that AI-assisted planning produced significantly lower angular and linear deviations, reduced planning time, and higher clinician confidence, while maintaining strong agreement with manual methods for basic morphometric measurements, such as bone height and width.

The slight reduction in bone height and buccolingual width observed with AI, although statistically significant, was clinically negligible. This difference may be attributed to algorithm-driven edge detection and segmentation techniques, which tend to produce more conservative and standardized measurements than manual interpretation. Manual measurements are inherently operator dependent and may be influenced by subjective landmark identification and variability in slice selection. Similar findings have been reported in previous studies, where AI-based systems demonstrated minor but consistent differences in linear measurements owing to improved reproducibility and reduced observer bias [11,12]. Ntovas et al. [13] showed that AI-based CBCT registration is as accurate as conventional methods under ideal conditions, although the accuracy may be influenced by artifacts and operator factors, supporting the reliability of AI observed in the present study.

A key finding of this study was the significantly lower angular and linear deviations observed in AI-assisted planning. Banerjee et al. [14] reported that computer-guided implant surgery demonstrates high accuracy, with reduced deviations compared to conventional approaches in clinical and in vitro settings. These findings support the improved positional accuracy observed with the AI-assisted planning in the present study. This suggests superior accuracy in predicting the implant position relative to the actual postsurgical outcome. The improved performance of AI can be explained by its ability to integrate three-dimensional anatomical data, maintain consistent reference axes, and eliminate human errors related to spatial judgment. In contrast, manual planning relies heavily on clinician experience and visual estimation, which may introduce variability [15]. Satapathy et al. [7] demonstrated that AI-assisted implant treatment planning showed comparable accuracy to clinician-generated plans with improved consistency and efficiency. These findings align with those of the present study, in which AI exhibited superior precision and reduced the planning time. These findings are consistent with previous investigations demonstrating the enhanced precision of AI-assisted implant planning systems compared with conventional methods [16,17].

Another important observation was the substantial reduction in the planning time with AI. AI-assisted planning requires approximately one-third of the time required for manual assessment, highlighting its efficiency in clinical workflow [18]. This can be attributed to the automation of measurements, real-time implant simulation, and pre-programmed algorithms that streamline decision making. Reduced planning time has important implications in high-volume clinical settings, where efficiency directly affects patient throughput and clinician workload. Previous studies have similarly reported significant time savings with digital and AI-assisted implant planning tools [19,20].

Clinician confidence scores were also significantly higher with AI-assisted planning. This may reflect the supportive role of AI in providing objective measurements, visual guidance, and decision support. AI systems can act as a second opinion, reducing uncertainty and enhancing decision making, particularly in complex cases. This aligns with the growing perception that AI is an adjunct rather than a replacement for clinical expertise [19]. Khaohoen et al. [20] highlighted that AI applications in implant dentistry can enhance diagnostic accuracy and clinical efficiency while emphasizing the need for cautious integration due to ethical and reliability considerations.

ICC analysis revealed excellent agreement between AI and manual methods for bone height and width, indicating that both approaches are reliable for basic morphometric assessment. However, moderate-to-poor agreement was observed for angular and linear deviations, suggesting that AI and manual methods differ substantially in positional accuracy. This discrepancy may reflect the inherent limitations of manual planning in accurately translating virtual plans into spatially precise implant positions. On the other hand, AI benefits from algorithmic consistency and precise geometric calculations, which enhance reproducibility.

The Bland-Altman analysis further confirmed good agreement for bone height measurements, with minimal bias and narrow limits of agreement. This finding supports the reliability of AI in performing routine anatomical measurements. Additionally, AI has demonstrated superior accuracy in implant dimension selection, particularly for implant length [21]. This may be due to its ability to simultaneously evaluate multiple parameters such as bone availability, anatomical constraints, and prosthetic requirements.

Clinical implications

The findings of this study have several important clinical implications. AI-assisted implant planning can significantly enhance accuracy and efficiency, thereby improving the treatment outcomes and reducing the risk of complications associated with improper implant positioning. A reduction in planning time can streamline clinical workflows, particularly in busy practices. Furthermore, increased clinician confidence suggests that AI can serve as a valuable decision support tool, especially for less experienced practitioners. AI may also contribute to the standardization of implant planning, reduce variability across clinicians, and improve the overall quality of care.

Limitations

Despite its strengths, this study had several limitations. The retrospective design limits the control over data quality and case selection. The sample size was relatively small, which may have affected the generalizability of the findings. Variability in CBCT scan quality and anatomical complexity could also have influenced the results. In addition, only one AI software platform was evaluated, and the findings may not be applicable to other systems. The study also did not assess intraoperative factors or long-term clinical outcomes, which are important for comprehensive evaluation of implant success. Finally, manual assessment was performed by a single clinician, which may have introduced operator bias despite calibration.

## Conclusions

Within the limitations of this retrospective study, AI-assisted implant planning demonstrated superior accuracy, efficiency, and consistency compared to manual clinician-based assessment. AI showed significantly reduced angular and linear deviations, markedly shorter planning times, and higher clinician confidence. Although both methods exhibited strong agreement for basic morphometric measurements, AI provided more precise positional outcomes. These findings suggest that AI can serve as a reliable adjunct to implant treatment planning, enhancing clinical decision making and workflow efficiency. However, it should complement, not replace, clinician judgment, particularly in complex cases in which patient-specific factors and clinical expertise remain essential.
